# ssJSD: A Fusion of Sparsity and Spatial Information for HiC Single-Cell Clustering

**DOI:** 10.64898/2026.09.14.751457

**Published:** 2026-10-21

**Authors:** Sang Wan Lee, Shili Lin

## Abstract

Single-cell high-throughput chromatin conformation capture (scHiC) enables profiling three-dimensional genome architecture at cellular resolution, providing insights into cell-to-cell variability and cellular functions. Recent frameworks utilize spatial interaction patterns to derive dissimilarity measures for downstream tasks such as cell clustering. However, the inherent sparsity and ultra-high dimensionality of scHiC contact matrices pose significant challenges. A central hurdle is that existing measures typically treat all zeros without distinction, failing to differentiate biologically meaningful structural zeros (SZs) from technical dropouts. Here, we introduce ssJSD (spatial and sparsity informed Jensen-Shannon Divergence), a computational framework designed to explicitly account for scHiC-specific sparsity patterns. By integrating band-wise contact frequency profiles with SZ-induced sparsity matrices, ssJSD leverages both spatial interaction patterns and biological absence of contacts. We adopted two complementary integration strategies: an early fusion approach that concatenates information into a single representation, and a late fusion approach that integrates JSD-based dissimilarities through diverse averaging methods. Through simulations and applications to human cell lines and prefrontal cortex data, we demonstrate that ssJSD improves clustering accuracy and effectively distinguishes cell types. Our study indicates that integrating SZ patterns is important for accurately quantifying cell-to-cell variability in 3D genomics.

## Full Text Availability

The license terms selected by the author(s) for this preprint version do not permit archiving in PMC. The full text is available from the preprint server.

